# Analysis of the citation impact of Brazilian researchers in oral pathology and oral medicine over two decades

**DOI:** 10.4317/medoral.26681

**Published:** 2024-08-01

**Authors:** Luiz Miguel Ferreira, Árlen Almeida Duarte de Sousa, João Pedro Santos Nascimento, Fabrício Emanuel Soares de Oliveira, Daniella Reis B Martelli, Eduardo Araújo de Oliveira, Hercílio Martelli-Júnior

**Affiliations:** 1 Department of Oral Diagnosis, School of Dentistry, University of Campinas, FOP-UNICAMP, Piracicaba, São Paulo, Brazil; 2 Postgraduate Program in Health Sciences, State University of Montes Claros, UNIMONTES, Montes Claros, Minas Gerais, Brazil; 3 Postgraduate Program in Primary Health Care, State University of Montes Claros, UNIMONTES, Montes Claros, Minas Gerais, Brazil; 4 Department of Pediatrics, Health Sciences Postgraduate Program, School of Medicine, Federal University of Minas Gerais, UFMG, Belo Horizonte, Minas Gerais, Brazil; 5 Department of Pediatrics, Rady Children’s Hospital, University of California, San Diego, United States; 6 Oral Pathology and Oral Medicine, School of Dentistry, State University of Montes Claros, UNIMONTES, Montes Claros, Minas Gerais, Brazil

## Abstract

**Background:**

Brazilian Oral Pathology (OP) and Oral Medicine (OM) have gained significant international recognition. However, no study has yet evaluated the impact of citations in scientific publications. Therefore, this study aimed to analyze the impact of citations from Brazilian researchers in OP and OM over the last two decades.

**Material and Methods:**

This was a cross-sectional study involving 50 researchers linked to postgraduate programs in OP/OM. Data collected from each professional's Lattes curriculum included gender, academic affiliation, the corporate category of the institution, and location. The number of papers published and citations received between 2004 to 2013 and 2014 to 2023 was also collected from the Web of Science database.

**Results:**

Most researchers were male (56%) and from public institutions (90%), mainly in the Southeast region (60%). Over two decades, they collectively published 8,033 scientific articles, with significant growth (*p*<0.001) from to 2004-2013 to 2014-2023. While the average citations per researcher did not differ significantly between 2004-2013 and 2014-2023 (*p*=0.538), there was a noTable 67.67% increase in citations in the last decade.

**Conclusions:**

Brazilian researchers in the areas of OP and OM have demonstrated a significant academic impact over the past two decades, with a marked increase in publications and citations over the last ten years. This highlights the contribution of Brazilians to the global scientific community in these areas.

** Key words:**Oral pathology, oral medicine, bibliometrics, work performance.

## Introduction

Bibliometrics, introduced in 1926, serves as an important tool for quantifying and analyzing scientific information, thus facilitating the identification and monitoring of research areas (1). The use of bibliometric indicators has gained importance in the evaluation of scientific activity (2), with the assessment of citations emerging as a prevalent method for evaluating academic and scientific performance (3). Consequently, Brazilian research agencies such as the Coordination for the Improvement of Higher Education Personnel (CAPES) (www.gov.br/capes/pt-br) and the National Council for Scientific and Technological Development (CNPq) (www.gov.br/cnpq/pt-br) emphasize the importance of quantitative indicators for understanding scientific dynamics, which are essential for guiding scientific policies and evaluating their effectiveness (4).

Despite the instability of financial support over the years, Brazilian dental research has achieved international recognition (5,6). Previous bibliometric analyzes have underscored the substantial contributions of the country to the fields of Oral Pathology (OP) and Oral Medicine (OM) (7,8,9). These studies demonstrated the country's strong presence in the field through its submission to indexed journals and its encouragement of international collaboration (7). Professionals in the areas of OP and OM also play fundamental roles by serving on editorial boards of academic journals, evaluating research support institutions, mentoring new researchers, and making significant contributions to scientific and technological advancement (8). Furthermore, according to CAPES, many Brazilian researchers are affiliated with postgraduate programs, contributing approximately 85% of the total national scientific production (www.gov.br/capes/pt-br).

Although updating the profile of production and researchers is essential to align scientific policies with the growth of national research (2), the challenge of monitoring quantity, quality, and temporal evolution persists in scientific contributions. High citation rates do not always correlate with quality or validity (10). However, publications and citations play a prominent role in building a researcher's prestige and success within the academic community (11).

Although Brazilian OP and OM have garnered significant international recognition, no study has evaluated the impact of citations in scientific publications. Thus, this study was designed to assess the influence of citations by Brazilian researchers on OP and OM. The findings of this study are anticipated to offer a more comprehensive understanding of the developmental trajectory of these research areas, underscoring the significance of these researchers' contributions to scientific progress.

## Material and Methods

- Design and Participants

This is a cross-sectional study. Researchers with scientific productivity scholarships from CNPq in the areas of OP and OM were included in this study (http://www.bi.cnpq.br/painel/mapa-fomento-cti/). Of the 230 researchers in dentistry, 36 had OP and OM as their main areas of investigation (12). In addition to these 36 researchers, postgraduate program advisors from both specialties were added, totaling to 62 researchers. However, 12 (19.35%) were excluded from the analyzes because of the following factors: death, retirement, withdrawal from research activities, and lack of updating the Lattes curriculum (https://lattes.cnpq.br/) for at least one year. Therefore, all evaluations were carried out with a database composed of 50 researchers, including CNPq research productivity fellows and/or researchers from postgraduate programs in OP/OM.

- Data collection

The curriculum vitae of each selected researcher was consulted through the CNPq Lattes platform (https://lattes.cnpq.br/) and used to extract the information needed for the study. The following information was collected from each professional: gender, academic affiliation, corporate category of the institution, geographical region, number of articles published, and number of citations received. To analyze the temporal evolution of academic production and the impact of researchers' studies over two decades, we evaluated two periods: between 2004 and 2013 and between 2014 and 2023. We consulted the Web of Science database (https://www. webofscience. com) to capture information on the H-index and calculate the m-quotient (3). The main variable of interest was the number of citations received by the researcher.

- Statistical analysis

The Statistical Package for Social Sciences (SPSS) version 27.0 for Windows® and GraphPad Prism 8® (GraphPad Prism Software Inc., San Diego, CA) was used to create the database and perform statistical analyses. Categorical variables were analyzed using relative and absolute frequencies, whereas means and their respective 95% confidence intervals were employed for the numerical variables. The Shapiro-Wilk test was used to assess data normality, while the Wilcoxon test was used to determine whether there was a significant difference between the means of articles and researchers' citations during the two time periods analyzed. A significance level of 5% (*p* ≤0.05) was considered.

## Results

Of the 50 participants in this study, 28 (56%) were male. The mean time since receiving the PhD was of 19.46 years (95% CI 16.84-22.08). Notably, the Southeast region of Brazil was the most prevalent, with 30 (60%) researchers originating from this area. Of these, 18 (36%) lived in the state of São Paulo, with 8 (16%) linked to the Faculty of Dentistry of Piracicaba at the University of Campinas and 8 (16%) linked to the University of São Paulo. Interestingly, 90% (*n*=45) of the home institutions represented by researchers were public institutions (Table 1).

Over two decades, the 50 Brazilian researchers collectively published 8,033 papers indexed in the WoS database, equating to an average of 160.66 (95% CI 128.21-193.11) scientific articles per researcher. Notably, there was a substantial increase of approximately 63% in the total number of publications from 2014 to 2023. Furthermore, a significant difference was observed when comparing the mean number of papers published between the periods 2004-2013 (776.96; 95% CI=523.15-1,030.77) and 2014-2023 (1,302.74; 95% CI=442.88-2,162.60) (*p*<0.001).

**Figure 1:**
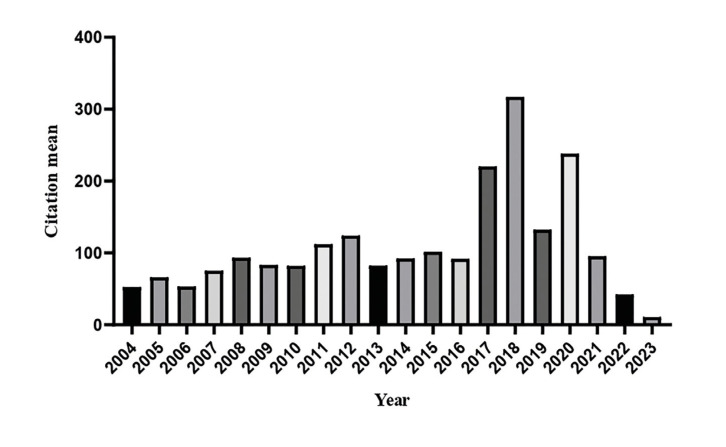
Mean citations received in WoS per year (2004-2023) by researchers (*n*=50) in the areas of Oral Pathology and Oral Medicine.

The researchers received a total of 103,985 citations in the WoS, with an average of 2,080.24 citations per researcher (95% CI=1,106.15-3,054.33) over a period of 20 years. In terms of annual citations per researcher, an average of 77.7 citations were obtained for the period from 2004 to 2013, and 130.27 citations for the years 2014 to 2023. When examining the average number of citations that researchers receive annually, it is evident that 2018, 2020, and 2017 were the most noTable years, as depicted in Fig. 1. Although there was no significant difference (*p*=0.538) in the average number of citations received between 2004-2013 and 2014-2023, there was a remarkable 67.67% increase in citations over the last decade (Table 2). The average number of citations per scientific article was also calculated, recording an average of 12.72 citations between 2004-2013, and 13.08 citations between 2014-2023.

**Figure 2:**
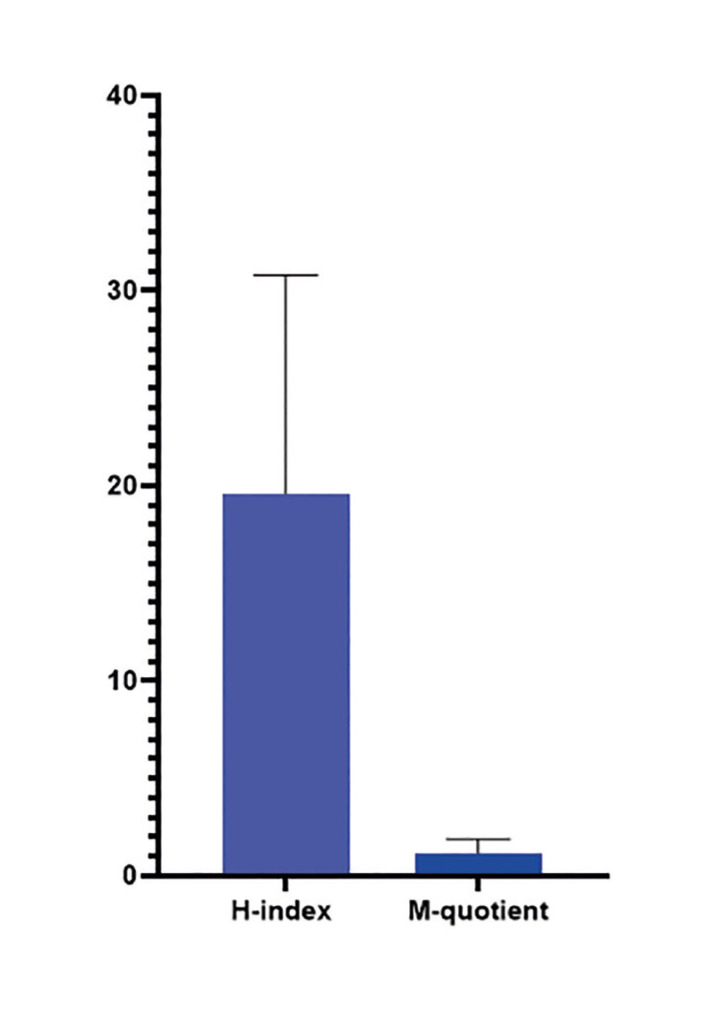
H-index and m-quotient representing the productivity and impact of researchers (*n*=50) in the areas of Oral Pathology and Oral Medicine.

The group's mean H index was 19.60 (95% CI=16.42-22.78), with a maximum value of 46 and a median of 20. As for the mean m-quotient, it was 1.164 (95% CI= 0.956-1.373), with a maximum value of 3.50 and a median of 1.047. This information is illustrated in the box plot in Fig. 2.

## Discussion

This study is the first to investigate the impact of scientific output from Brazilian researchers on OP and OM. Following Bellini's (13) proposal, we examined the most relevant parameters to assess the quality of scientific production, encompassing the total number of papers, citations, and average citations per article. Thus, we provide pertinent and up-to-date data on indicators related to scientific publications and citations by comparing professionals at two distinct time points. As anticipated, our analysis demonstrated that these professionals were highly scientifically productive and had received a high number of citations.

Traditionally, the publication and citation of research results has been fundamental in justifying the advancement, promotion, and maintenance of academic positions. The quantity and merits of publications are considered indicators of academic competence. The frequency with which a work is referenced is also significant because it highlights the relevance and influence of research in the field (11). Furthermore, the analysis of various research elements in this domain can guide future investigations, contribute to the planning of development in various areas of Dentistry, optimize the distribution of financial and physical resources, and enhance both the quantity and quality of production in this field (14).

Our analysis revealed a predominance of male researchers (1.27:1). Despite women's involvement in research, their representation in the scientific community in general is still limited to only one-third (33.3%) in the last UNESCO science report (https://unesdoc.unesco.org/ark:/48223/pf0000235406). As noted by Handelsman *et al*. (15), women still have a history of low representation in science, which is reflected in the persistence of gender inequality. Shen (16) supports this view by pointing to discrimination, wage gaps, and resource limitations faced by women in the scientific community. This reality is also evident in the dental context, where despite the increase in the number of female students, leadership positions and prominent opportunities are still predominantly held by males (17). Furthermore, a study by Sartori *et al*. (18) highlighted the persistence of male dominance in publications in high-impact journals, resulting in lower female representation as authors in high-impact studies.

The geographical distribution of the analyzed researchers was heterogeneous, with 60% of them being in the Southeast region of the country, while the North region had no researchers. This panorama has also been identified in other studies, which describe the predominance of researchers, mainly in the states of São Paulo and Minas Gerais, and affiliated with educational institutions, such as the University of Campinas and the University of São Paulo (8). Among the 70 higher education institutions offering postgraduate programs in Dentistry in Brazil, nearly half of them, 31 (44.28%), are in the Southeast region. Consequently, most master's and PhD programs are concentrated in this region. It is also important to highlight that postgraduate programs with the highest grades (six and seven) are situated in the Southeast of the country (https://sucupira.capes.gov.br/sucupira/). These factors may explain why many researchers have worked in this geographic region.

Citation analysis can be affected by temporal bias, as scientific articles typically receive citations progressively over time. In this sense, recent articles may not have had enough time to accumulate citations (13). However, in the present study, there was no significant difference (*p*=0.538) between the citations received in the periods 2004-2013 and 2014-2023. Additionally, approximately 63% of the total citations were received between 2014 and 2023 compared to the previous decade. Therefore, it is important to consider other factors that may influence citation analysis, such as the research area, visibility of the journal where the article is published, and impact of the authors involved.

The publication language can also influence the choice of studies by authors to support their research, with a clear preference for articles published in English-language scientific journals. Additionally, there is an inherent bias toward the age of the studies, as any works published in the 20th century are subject to exclusion, which may result in the exclusion of articles considered classical (19). Moreover, this type of citation analysis often does not consider self-citation, citations in textbooks, or references made in lectures, which may result in an underestimation of the actual number of citations received by scientific articles (20).

Another important variable in our study was the H-index, for which we obtained a mean value of 19.6, which is similar to the value obtained in our previous dentistry study (8). The H-index is a parameter that evaluates both the quantity and relevance of citations to a researcher's work, providing a single number that summarizes the research output and its impact (11,21). This feature helps balance the influence of both quantity and quality on a researcher's overall research productivity (22).

However, the H-index has limitations and is often criticized for its tendency to favor researchers with a long-track record in the field, as they have had more time to publish and accumulate citations. This bias can disadvantage early career researchers who have not had enough time to produce or gain recognition in terms of citations. Additionally, the H-index can be manipulated through excessive self-citation, in which researchers repeatedly cite their own work to artificially increase the number of citations (23). Another limitation of this parameter is that it does not consider the specific number of citations of each article beyond the minimum necessary to achieve a certain H-index. For example, a researcher with 30 works, each cited 100 times, will have an H-index of 30, similar to another researcher with 30 works, each with 30 citations, who will also have an H-index of 30 (11).

One way to compare scientists and reduce the bias associated with career time is to calculate the H-index and divide it by the number of years they have been active in research (24). Hirsch (3) suggested dividing the H-index by the number of years since a scientist's first publication, calling this result the m-quotient. Therefore, in our study, we calculated the m-quotient of the researchers and obtained a mean value of 1.164. This value of approximately m equals 1 (which is equivalent to an H-index of 20 after 20 years of scientific activity) is indicative of a successful scientist (3). This result corroborates the statement that this group of Brazilian researchers analyzed has an impact on the scientific community, even when the time factor is considered in the calculation.

In conclusion, our results reveal a significant academic impact, with a noTable increase in both publications and citations over the last decade among researchers in the areas of OP and OM. Despite the inherent limitations of citation analysis, our study highlights the substantial contribution of Brazilian researchers to the global scientific community in these areas. Furthermore, it emphasizes the importance of considering factors such as research area, journal impact factor, and popularity of the authors involved, as they can also influence the number of citations.

## Figures and Tables

**Table 1:** Characterization of researchers in the areas of Oral Pathology and Oral Medicine (n=50).

Variables		n	%
Gender	Female	22	44
	Male	28	56
State	São Paulo	18	36
	Minas Gerais	9	18
	Pernambuco	5	10
	Rio Grande do Norte	4	8
	Paraíba	4	8
	Rio de Janeiro	3	6
	Paraná	2	4
	Bahia	2	4
	Distrito Federal	1	2
	Goiás	1	2
	Rio Grande do Sul	1	2
Region	Midwest	2	4
	Northeast	15	30
	North	0	0
	South	3	6
	Southeast	30	60
Researcher's institution	University of Campinas	8	16
	University of São Paulo	8	16
	Federal University of Minas Gerais	5	10
	Federal University of Pernambuco	3	6
	Federal University of Rio Grande do Norte	3	6
	State University of Montes Claros	3	6
	Federal University of Paraíba	2	4
	Federal University of Rio de Janeiro	2	4
	João Pessoa University Center	2	4
	State University of Western Paraná	2	4
	AC Camargo Cancer Center	1	2
	Federal University of Alfenas	1	2
	Federal University of Bahia	1	2
	Federal University of Goiás	1	2
	Federal University of Rio Grande do Sul	1	2
	Maurício de Nassau University Center	1	2
	Nova Esperança University	1	2
	Oswaldo Cruz Foundation	1	2
	Private Clinic	1	2
	Rio de Janeiro State University	1	2
	University of Brasília	1	2
	University of Pernambuco	1	2
Institutional corporate format	Public	45	90
	Private	5	10
Postdoctoral	Yes	29	58
	No	21	42

**Table 2:** Scientific publication by professionals in the areas of Oral Pathology and Oral Medicine (n=50).

Variables	Mean	IC 95%	n total	*p-Value*
Published articles (2004-2013)	61.08	46.98-75.18	3,054	0,001*
Published articles (2014-2023)	99.58	78.68-120.48	4,979	
Articles citations in WoS (2004-2013)	776.96	523.15-1,030.77	38,848	0.538
Articles citations in WoS (2014-2023)	1,302.74	442.88-2,162.60	65,137	

Note: The significance of the difference between means was measured by the Wilcoxon test. *Significant *p-value* < 0.05.
